# Phase states, microstructure and dielectric characteristics of solid solutions (1 – *x*)NaNbO_3_ – *x*Ca_2_Nb_2_O_7_ and (1 – *x*)NaNbO_3_ – *x*Sr_2_Nb_2_O_7_

**DOI:** 10.1016/j.heliyon.2020.e05197

**Published:** 2020-10-24

**Authors:** J.Y. Zubarev, S.-H. Chang, C. Lin, N.A. Boldyrev, A.V. Pavlenko, A.V. Nazarenko, A.V. Nagaenko, Y.I. Yurasov, I.A. Verbenko, I.A. Parinov, L.A. Reznichenko

**Affiliations:** a Research Institute of Physics, Southern Federal University, Rostov-on-Don, Russia; b National Kaohsiung University of Science and Technology, Department of Marine Environmental Engineering, Kaohsiung, Taiwan; c Southern Scientific Center of the Russian Academy of Sciences, Rostov-on-Don, Russia; d Institute for Advanced Technologies and Piezotechnics, Southern Federal University, Rostov-on-Don, Russia; e Vorovitch Research Institute of Mechanics and Applied Mathematics, Southern Federal University, Rostov-on-Don, Russia

**Keywords:** Materials science, Materials chemistry, Layered perovskite-like compounds, Ceramics, Calcium pyroniobate, Water intercalation, Phase diagram, Dielectric characteristics

## Abstract

Ceramics of binary systems solid solutions (1 – *x*)NaNbO_3_ – *x*Ca_2_Nb_2_O_7_ and (1 – *x*)NaNbO_3_ – *x*Sr_2_Nb_2_O_7_ with non-isostructural extreme components were prepared by the solid-phase reactions technique with the following sintering using conventional ceramic technology. It was found that ceramics with *x* ≤ 0.2 have a perovskite structure. Layered type of structure predominates in the concentration range 0.2 < *x* ≤ 1. Phase diagrams of both systems at room temperature have been determined in the perovskite area. It was shown that this area contains two concentration regions with the different crystal structures and the morphotropic phase boundary between them. Microstructure and dielectric characteristics of selected solid solutions were investigated. The influence of technological regulations, such as mechanical activation and variation of sintering temperatures, on the formation of the microstructure and dielectric characteristics was studied for the individually selected concentrations (*x* = 0.1 and *x* = 0.25). Dielectric characteristics of ceramics revealed the presence of the Maxwell-Wagner polarization and its corresponding relaxation in the solid solutions (1 – *x*)NaNbO_3_ – *x*Ca_2_Nb_2_O_7_ at x > 0.20.

## 1 Introduction

Recently, the perovskite-like layer structured (PLS) ferroelectrics with a formula of *A*_*n*_*B*_*n*_O_3*n*+2_ have received considerable attention because of their wide ranging properties. The properties of *A*_*n*_*B*_*n*_O_3*n*+2_ are related to their unique structure composed of *BO*_*6*_ octahedra with *A* cations within the perovskite-like layers, where *n* is the number of octahedral layers [1, 2]. Ferroelectrics Ca_2_Nb_2_O_7_ (CN) and Sr_2_Nb_2_O_7_ (SN) are representatives of this class of materials and currently being considered as the basis for many applications. CN is considered as a promising compound for non-linear optics [3], laser technology [4], highly active photocatalyst for water splitting [5, 6], design a multifunctional optical storage device [7, 8]. Both the CN and SN demonstrate mild piezoelectric properties (*d*_*33*_ < 10 pC/N) with extremely high Curie temperatures (~1800 K and ~1600 K, respectively) [9, 10]. These materials possess very high electrical resistance which decreases with the temperature due to electronic/ionic conduction at elevated temperature [2, 11, 12]. This suggests that CN and SN can found broad application in the aerospace, automotive, and power generating industries [1, 10]. In the past years, appreciable attention has been given to enhance properties of the PLS ferroelectrics by modification [4, 13, 14, 15] and creating new solid solutions [16, 17, 18]. In this work samples of the binary systems solid solutions (1 – *x*)NaNbO_3_ – *x*Ca_2_Nb_2_O_7_ (NCN) and (1 – *x*)NaNbO_3_ – *x*Sr_2_Nb_2_O_7_ (NSN) were the objects of study. The second component of these systems antiferroelectric NaNbO_3_ is often used in development of the lead-free industrial piezoceramics. The presence of non-isostructural components (NaNbO3 and pyroniobates) can lead to the appearance of the new phases with unique characteristics and morphotropic phase boundaries (MPB). It is known that MPB in different multiferroic and ferroelectric solid solutions can significantly enhance the performances of the materials [19, 20, 21]. At the same time, production technological regulations (variations in sintering temperature and mechanical activation) also have a strong influence on the phase composition and characteristics of the obtained ceramics. Therefore this work aimed to investigate phase composition, crystal structure, microstructure and dielectric characteristics of NCN and NSN systems and to evaluate the influence of technological regulations on the macroresponces of the obtained solid solutions.

## 2 Experimental

The binary solid solution systems (1 – *x*)NaNbO_3_ – *x*Ca_2_Nb_2_O_7_ and (1 – *x*)NaNbO_3_ – *x*Sr_2_Nb_2_O_7_ were investigated in concentration region 0 ≤ *x* ≤ 1.00 (0 ≤ *x* ≤ 0.2, *Δx* = 0.025 and 0.2 < *x* ≤ 1.00, *Δx* = 0.10). Ceramic samples were obtained using conventional ceramic technology by double solid-phase synthesis at temperatures *T*_*1*_ = (1220÷1250) K and *T*_*2*_ = (1370÷1470) K depending on composition and holding times *τ*_*1*_ = *τ*_*2*_ = 4 h with following sintering at *T*_*Sin*_ = (1530÷1660) K during 2.5 h NaHCO_3_, Nb_2_O_5_, CaCO_3_, SrCO_3_ with the content of the main substance not less than 99.9% were the initial reagents. Samples for sintering were pressed in the form of disks with a diameter of 10 mm and a thickness of 1 mm. After polishing, electrodes were deposited onto flat surfaces of the disks by stepwise firing of the silver paste. Samples with *x* = 0.10 and *x* = 0.25 of both systems were also prepared using mechanical activation to compare their microstructure and macroresponses with other samples (NCN10, NCN25, NSN10, NSN25) (Table 1). Mechanical activation of synthesized powders was carried out using planetary ball millAGO-2. The compositions with the balls of ZrO_2_ with a diameter of 8 mm and a total mass of 200 g were loaded into cylinders with an internal diameter of 63 mm. The cylinders with the mixture were placed in a mill, where the powders were mixed up in an alcohol medium for 10 min at a drum rotation speed of 1800 rpm.

**Table 1:** Densities of the not mechanoactivated and mechanoactivated NCN and NSN solid solutions.

Objects	not mechanoactivated			mechanoactivated		
*Т*_Sin_, К	1520	1570	1620	1520	1570	1620
density	*ρ*_exp_, *g*/cm^3^	*ρ*_exp_, *g*/cm^3^	*ρ*_exp_, *g*/cm^3^	*ρ*_exp_, *g*/cm^3^	*ρ*_exp_, *g*/cm^3^	*ρ*_exp_, *g*/cm^3^
NCN10	4.46	4.48	4.49	4.2	4.5	4.51
NSN10	4.37	4.44	4.5	3.89	4.47	4.44
NCN25	4.15	4.31	4.44	3.85	4.59	4.61
NSN25	4.22	4.39	4.39	4.31	4.45	4.45

X-ray powder diffraction studies were carried out using a diffractometer DRON-3 (Bragg-Brentano focusing, filtered Co_Kα_-radiation). In the interval 0.0 ≤ *x* ≤ 0.20 the parameters of the perovskite monoclinic cell (*a* = *c*≠*b*, α = γ = 90^o^≠β) were calculated using a quadratic form (1) [22]:

(1) $$d_{hkl}=\frac{a\;\sin\text{β}}{\sqrt{N}}\left(1+\frac{k^{2}}{N}y+\frac{lh}{N}\cos\text{β}\right)$$

where *N =h*^2^*+k*^2^*+l*^2^, *h*, *k*, *l* – diffraction indices, *y*+1 = *b/*(*a*sin*β*). Cell parameters were calculated by the X-ray peaks 200, 020, $\bar{2}02$ . The measurement errors of the structural parameters had the following values: Δ*a* = Δ*b* = Δ*c* = ±0.004 Å, Δβ = ±0.05^o^, Δ*V* = ± 0.10 Å^3^. To determine the cell multiplicity, experimental X-ray diffraction patterns were compared with theoretical ones computed using original programs.

The experimental density of the ceramics was determined by the method of hydrostatic weighing in octane. The study of the ceramic grain structure was carried out using the KEYENCE VK-9700 color laser scanning 3D microscope.

The study of the ceramics microstructure was carried out using KEYENCE VK-9700 scanning laser microscope. The source of light was a laser with a wavelength of 408 nm. The laser scanning resolution is 2048 × 1536 pixels with a 16-bit photomultiplier. The images were obtained by the method of confocal microscopy. Scanning electron microscope JSM-6390L (JEOL) with a resolution of 1.2 nm at an accelerating voltage of 30 kV was used for more detailed study of the samples microstructure.

Temperature dependences of complex dielectric permittivity *ε∗* = *ε*' – *iε*'' (*ε*′ and *ε*'' are the real and imaginary parts of *ε*∗, respectively) were measured at *T* = (300 ÷ 1000) K in the frequency range *f* =(10^−3^ Hz-1 MHz) using impedance analyzers Agilent 4285A, Agilent 4980A and RLC meter E7-20.

## 3 Results and discussion

**Figure 1:**
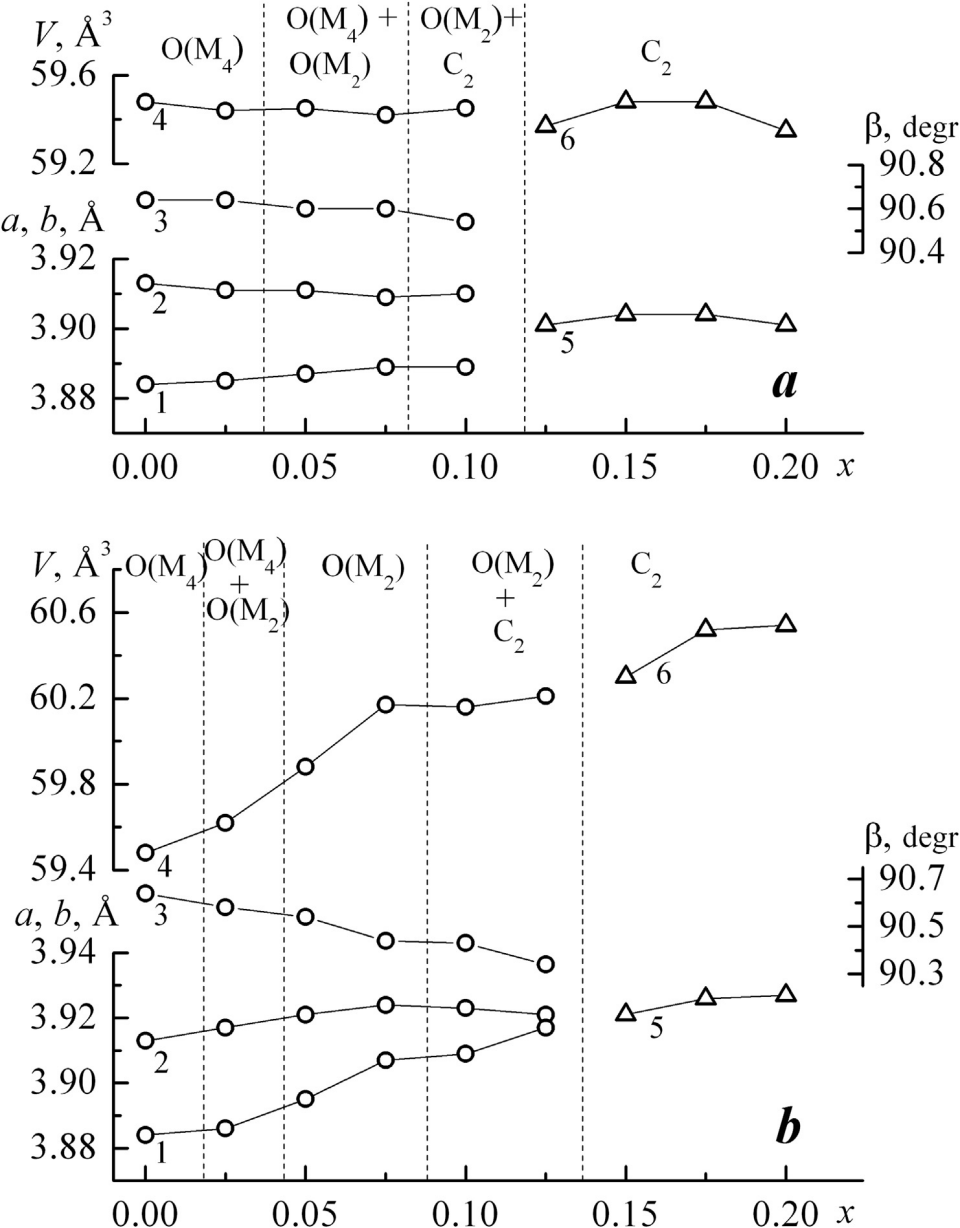
Concentration dependences of the perovskite cell parameters: *b*_*m*_ (1), *a*_*m*_ = *c*_*m*_ (2), *β* (3), *V*_*m*_ (4), *a*_*c*_ (5), *V*_*c*_ (6) of the NCN (a) and NSN (b) ceramics. Dashed lines indicate phase regions of different symmetries and multiplicities.

**Figure 2:**
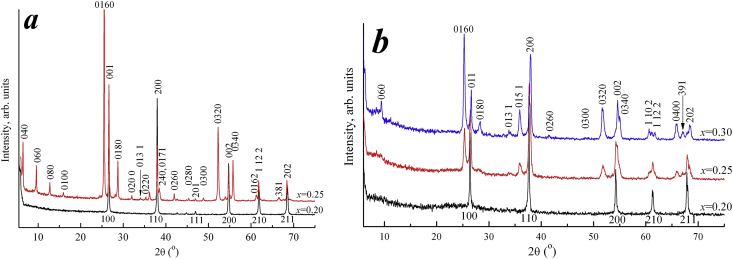
X-ray diffraction patterns of NCN samples with *x* = 0.2 and 0.25 (a) and NSN with *x* = 0.2, 0.25, 0.30 (b), the lower indices refer to the perovskite axes.

It was found (Figure 1) that solid solutions based on NaNbO_3_ with a perovskite (P) structure are formed in the concentration range 0.00 ≤ *x* ≤ 0.20 in both systems. The symmetry with increasing *x* changes from orthorhombic with a quadruple monoclinic subcell O (M_4_) to cubic with a superstructure (C_2_). Phase transformations occur in sequence O (M_4_) → O (M_4_ + M_2_) → O (M_2_) → O (M_2_) + C_2_ → C_2_. Two areas of coexistence of phases with different symmetry are established. The first of them is characterized by the coexistence of monoclinic subcells with different multiplicity. The transition from the orthorhombic type of symmetry to cubic is observed in the second concentration range. According to [23], at *x* > 0.2 (P) structure replaced by layered (L(*n*)) with different values of *n*. In general, both NCN and NSN systems have almost the same phase diagrams. Minor differences between them include the absence of a pure orthorhombic phase with a doubled monoclinic subsell in the NCN and constant increase of the parameters and volume of the unit cell in the NSN. Presented X-ray diffraction patterns of NCN samples with *x* = 0.2 and 0.25 (Figure 2a) and NSN with *x* = 0.2, 0.25, 0.30 (Figure 2b) shows the transition from the (P) structure to the PLS. It can be seen that NCN25 PLS compound is well structured, while NSN system structure was finally formed only at *x* = 0.3.

**Figure 3:**
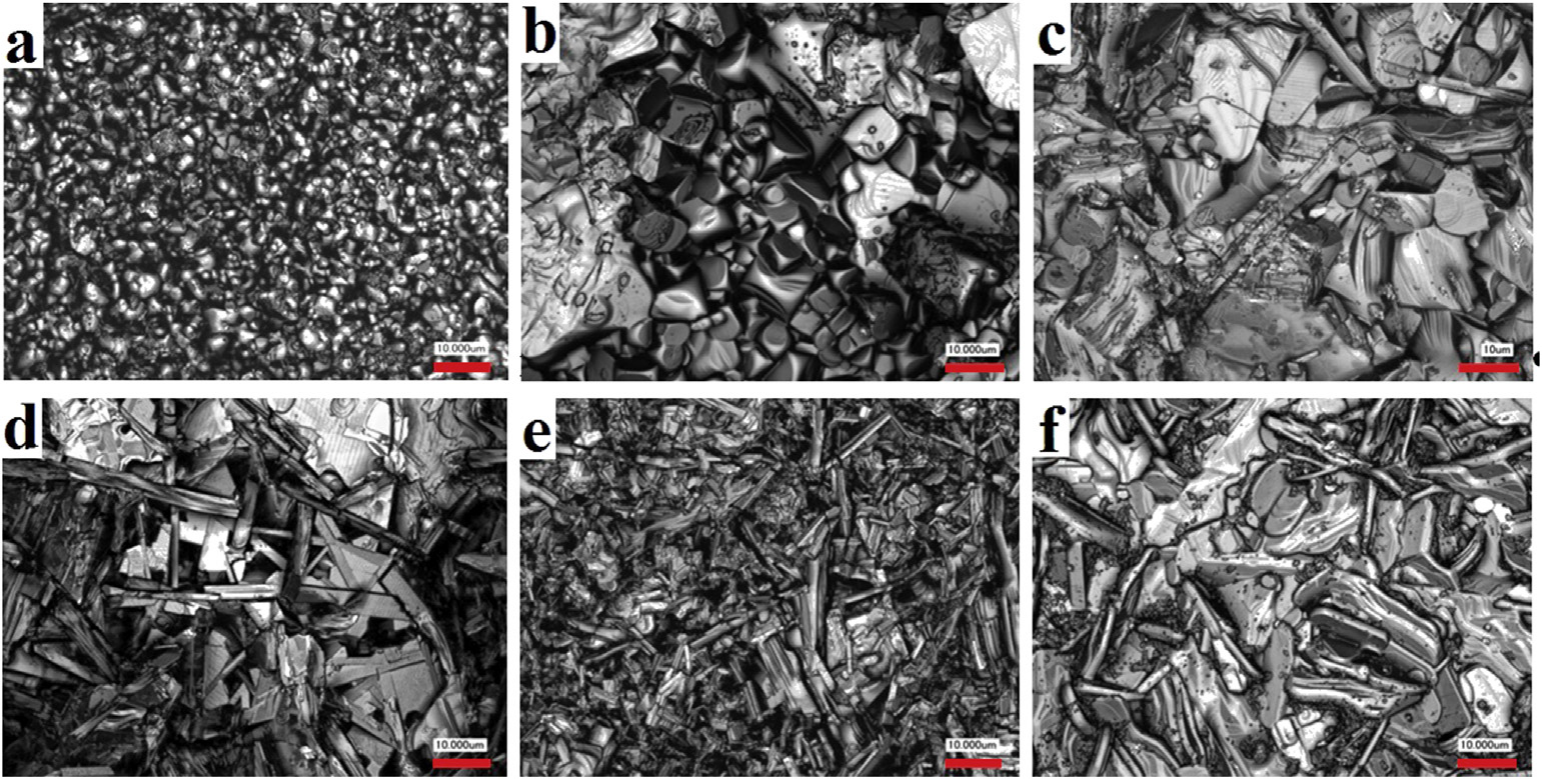
Fragments of the microstructure of the NCN solid solutions (a: *x* = 0, b: *x* = 0.175, c: *x* = 0.25, d: *x* = 0.3, e: *x* = 0.55, f: *x* = 1).

**Figure 4:**
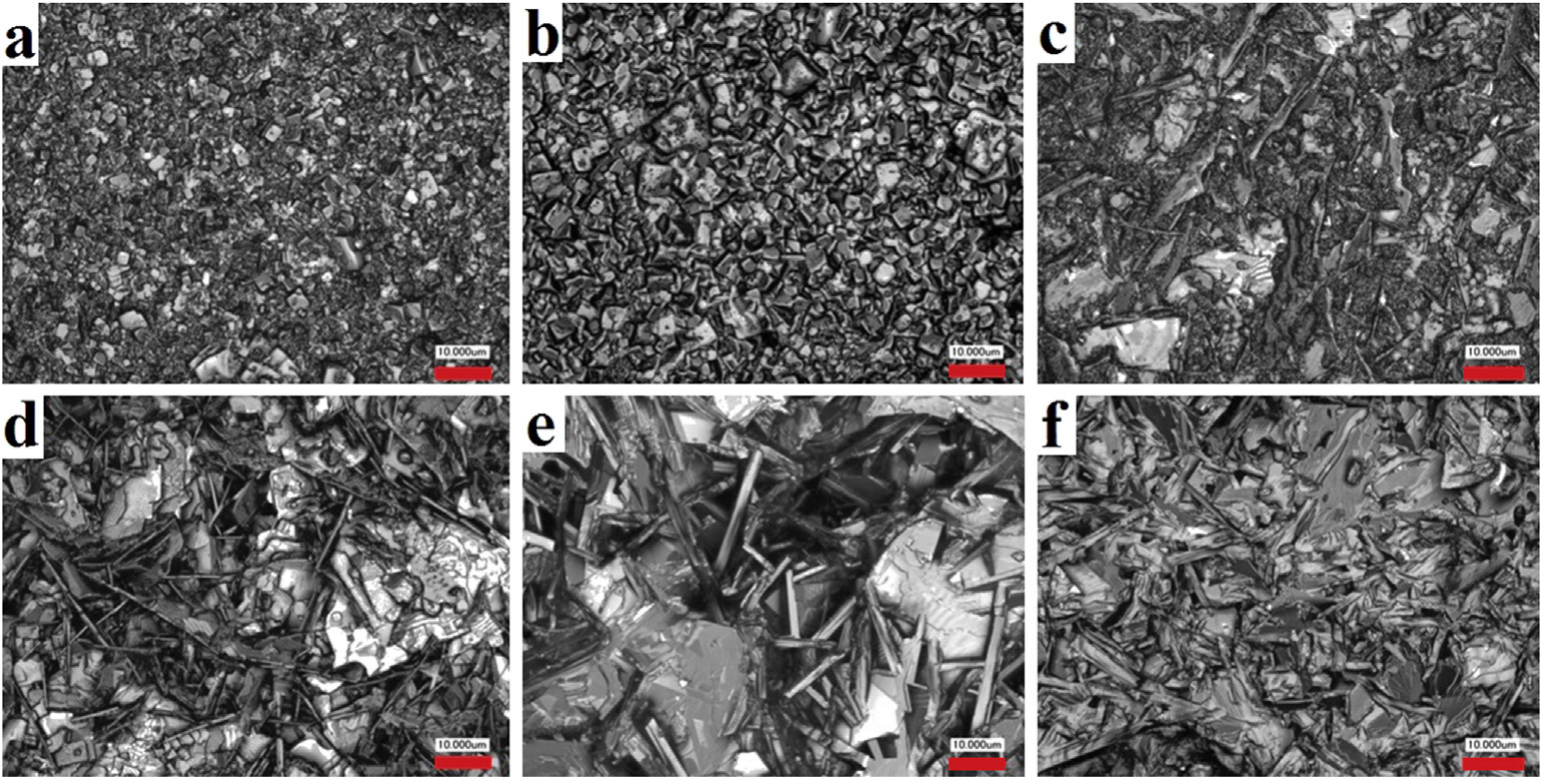
Fragments of the microstructure of the NSN solid solutions (a: *x* = 0.05, b: *x* = 0.15, c: *x* = 0.2, d: *x* = 0.25, e: *x* = 0.3, f: *x* = 1).

Figures 3 and 4 show fragments of microstructures of the NCN and NSN solid solutions, respectively, including basic compounds NaNbO_3_ (Figure 3a), CN (Figure 3f) and SN (Figure 4f). NaNbO_3_ has an isometric type of grain structure with crystallites having the shape of a cube. In general, the grain boundaries are thin. Crystallites packing is heterogeneous, grain size varies from 2 μm to ~20 μm. Similar grain structure is observed in the samples of both systems in the (P)-region of the phase diagram (Figures 3a, 3b, 4a, 4b). Further increasing *x* leads to the change of the type of polycrystallinity. Microstructure becomes anisometric with chaotic oriented grains of acicular and lamellar forms (Figures 3e, 3f, 4e, 4f), which is typical for layered compounds. It correlates with the evolution of the phase composition of the studied systems. The transition from the isometric to the anisometric type of the grain structure occurs in the intermediate concentration range (0.2 < *x* < 0.3) where crystallites of both types coexist (Figures 4c-4e).

**Figure 5:**
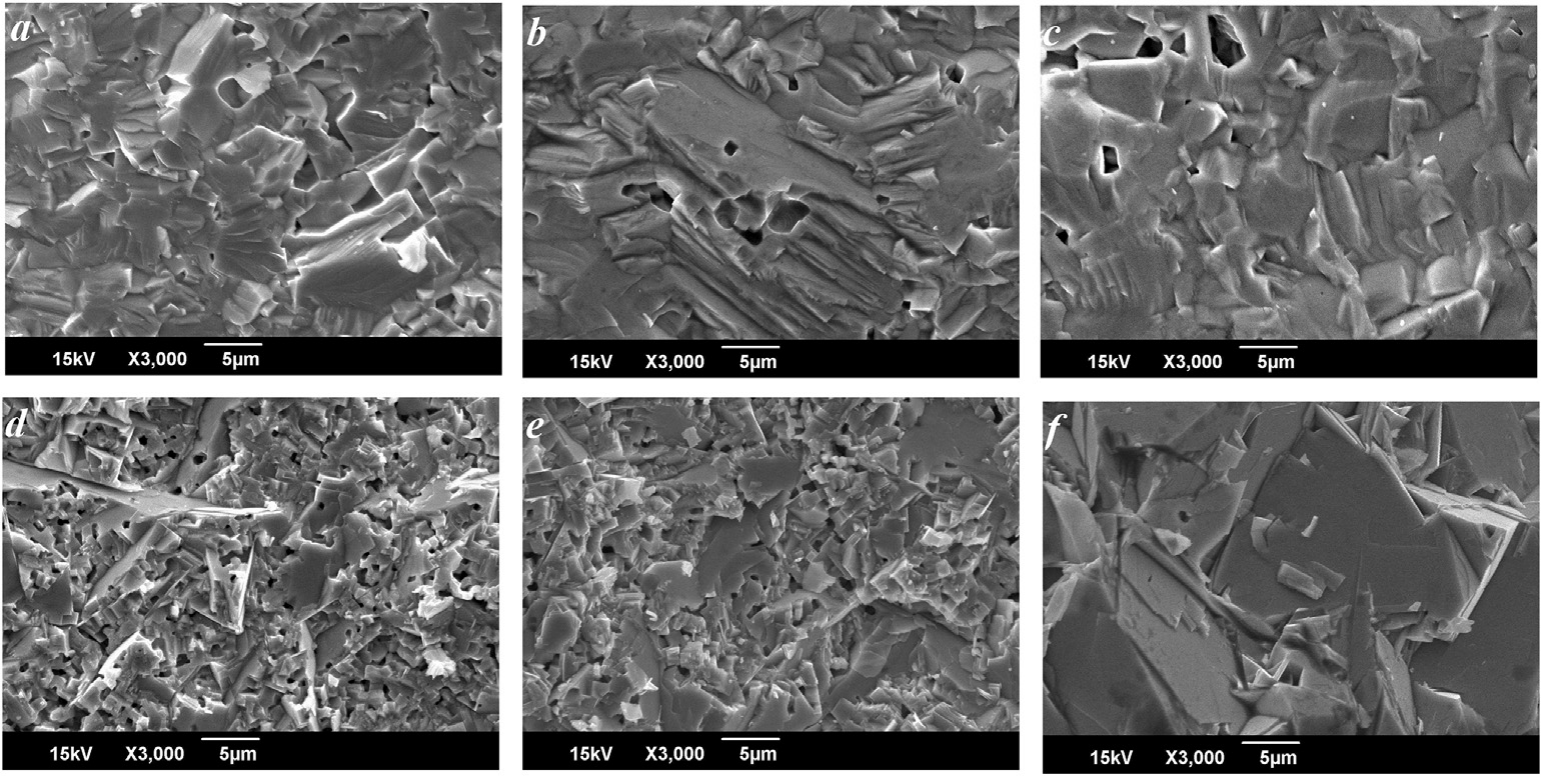
Fragments of the microstructure of the NCN ceramics obtained at various *T*_*Sin*_: *x* = 0.10 (a–c); *x* = 0.25 (d–f); (a, d) *T*_*Sin*_ = 1520 K; (b, e) *T*_*Sin*_ = 1570 K; (c, f) *T*_*Sin*_ = 1620 K.

**Figure 6:**
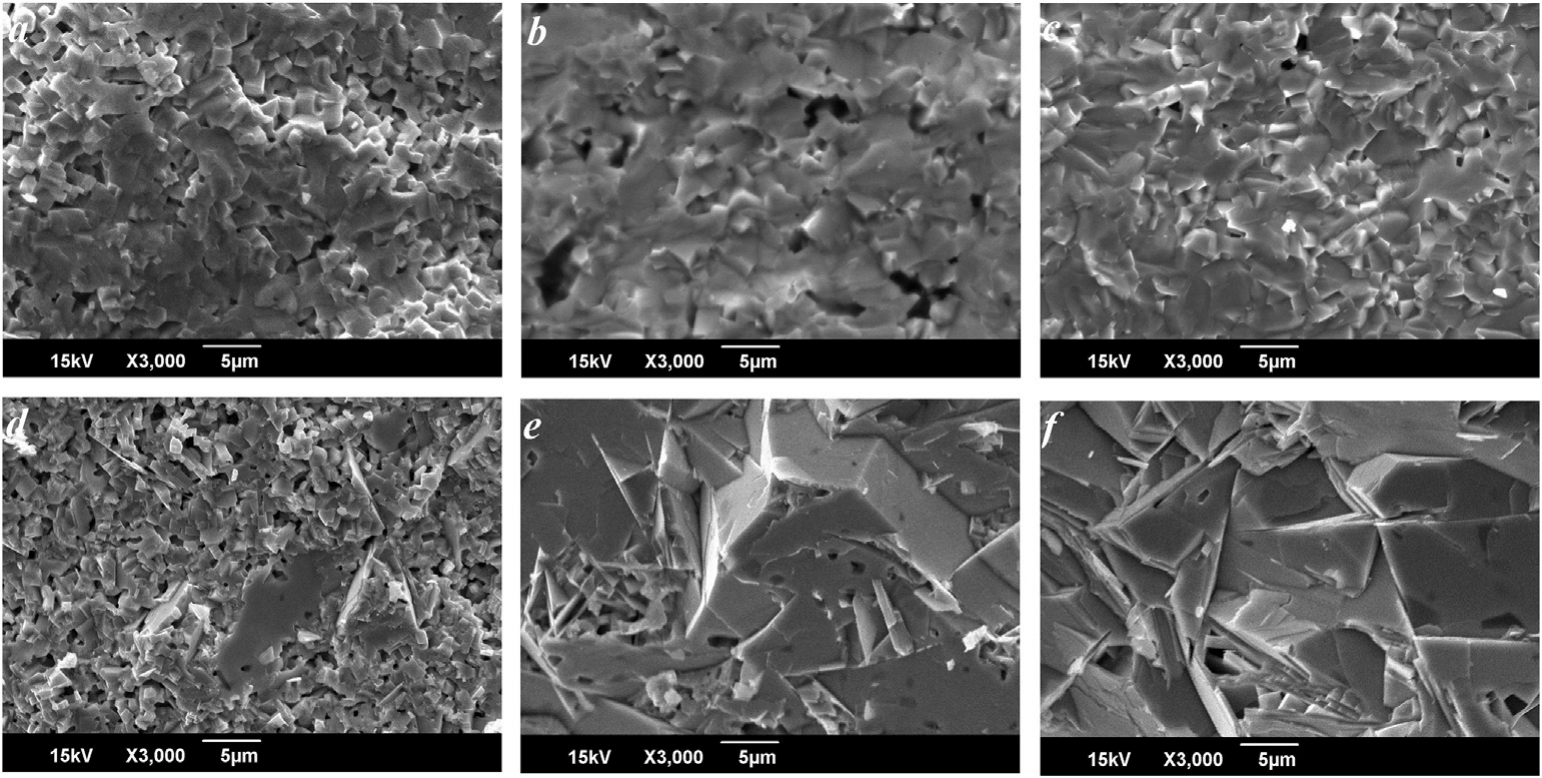
Fragments of the microstructure of the NSN ceramics obtained at various *T*_*Sin*_: *x* = 0.10 (a–c); *x* = 0.25 (d–f); (a, d) *T*_*Sin*_ = 1520 K; (b, e) *T*_*Sin*_ = 1570 K; (c, f) *T*_*Sin*_ = 1620 K.

Micrographs of the chips of NCN10, NCN25, NSN10 and NSN25 samples obtained at various *T*_*Sin*_ are presented on Figures 5 and 6. Both in NCN and NSN increasing *T*_*Sin*_ from 1560 K to 1620 K leads to the grain consolidation. It is associated with the activation of diffusion processes and mass transfer at the high temperatures during recrystallization sintering of ceramics. In the NCN10 ceramics from (P)-region of the phase diagram at low *T*_*Sin*_ = 1520 K microstructure with bulk grains is observed (Figure 5a). But further *T*_*Sin*_ increase leads to the formation of the needle-shaped grains (Figures 5b, 5c). It can be caused by presence of the clusters of the layered phase in the (P)-area in NCN solid solution. In solid solutions NSN10 the isotropy of grains is retained over the entire range of *T*_*Sin*_ (Figures 6a-6c). In NSN25 located between the perovskite and layered phases, a structure with isotropic grains forms at low *T*_*Sin*_ (Figure 6d). A further *T*_*Sin*_ rise leads to a significant increase in the average size of grains and a change of their shape to lamellar (Figures 6e, 6f). The difference between the recrystallization processes of NCN10 and NSN10 ceramics cam be explained by the crystal-chemical features of Ca-containing solid solutions. Unlike strontium cations, the small-sized Ca-cation can be localized in both the A- and B-positions of the perovskite structure that ensures its instability and leads to the formation of clusters of new phases in the NCN10. Mechanical activation has led to the rapid growth of crystallites to gigantic sizes in the NSN25 ceramics. At low *T*_*Sin*_, mechanical activation has led to the appearance of porosity and low density of samples (Table 1). However, at high *T*_*Sin*_, the density of mechanically activated ceramics was higher than that of non-mechanically activated analogs. This can be explained by the enhanced formation of primary recrystallization centers during mechanical activation. Moreover, at low *T*_*Sin*_ these centers interact with each other weakly, which leads to the formation of structures with weakened grain boundaries and, as a result, to friability and reduced ceramic density.

**Figure 7:**
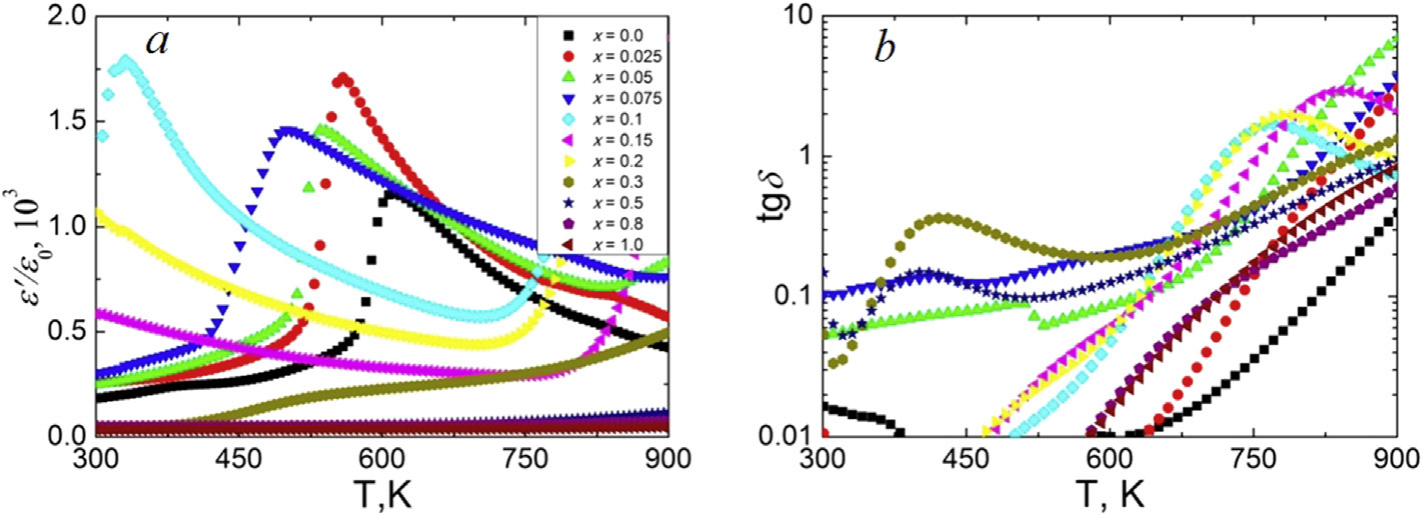
Temperature dependences of *ε'/ε*_*0*_ (a) and *tgδ* (b) at 100 kHz for NCN solid solutions.

**Figure 8:**
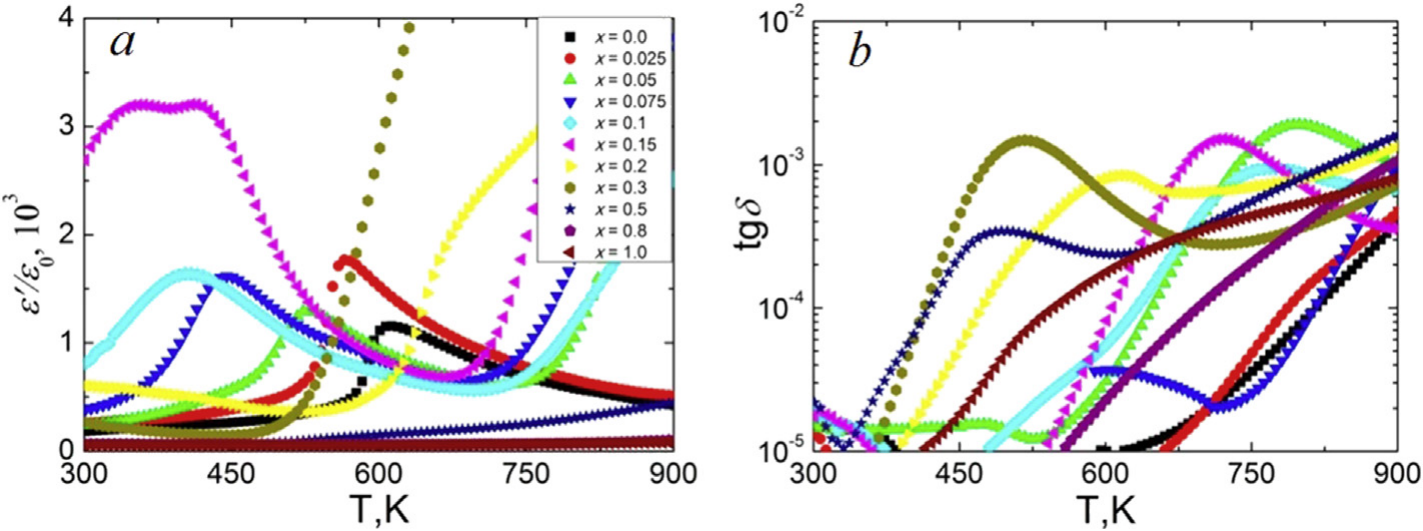
Temperature dependences of *ε'/ε*_*0*_ (a) and *tgδ* (b) at 100 kHz for NSN solid solutions.

**Figure 9:**
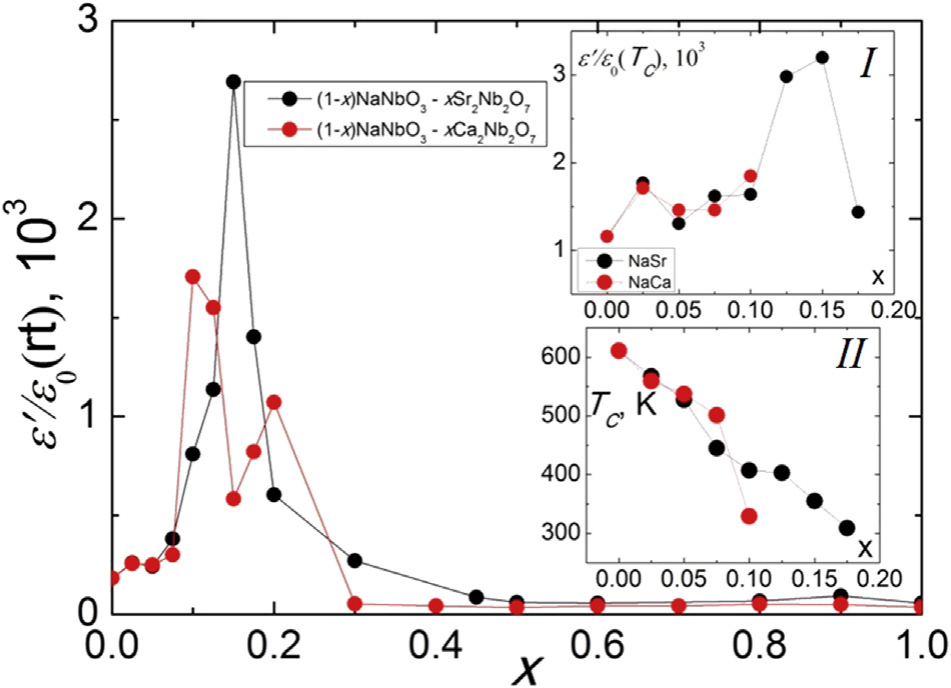
Dependences of *ε*′/*ε*_0(RT)_ (*x*), *ε*′/*ε*_0(*Tc*)_ (*x*) (inset I) and *T*_*C*_(*x*) (inset II) for NCN and NSN solid solutions.

Temperature dependences of the real part of the relative complex permittivity *ε'/ε*_*0*_ and dielectric loss tangent *tgδ* at 100 kHz for NCN are presented on Figure 7. Analysis of the dependences *ε'/ε*_*0*_(*T*) and *tgδ* (*T*) in NCN ceramics showed that samples can be divided into two groups. The first group includes compositions from (P)-region with *x* ≤ 0.2, which exhibit typical ferroelectrics (antiferroelectrics) *ε'/ε*_*0*_ behavior with peaks shifting to the lower temperature region with increasing *x*. The second group includes compositions with *x* > 0.2, where monotonic increasing *ε'/ε*_*0*_ is observed in the studied temperature range. This separation is caused by crystal structure differences between the solid solutions with *x* ≤ 0.2 and *x* > 0.2. Similar regularities are observed in NSN solid solutions (Figure 8). However, in addition to the shift of the *T*_*C*_ to the lower temperature region with increasing *x*, a stronger temperature blurring of the phase transition is observed. It may be associated with an increase in the crystal-chemical disorder in the studied ceramics. NSN solid solutions also demonstrate very low values of the loss tangent, which is three orders of magnitude lower than that in the NCN system. Analysis of the concentration dependences of *ε'/ε*_*0*_ at room temperature (Figure 9) and *T*_*C*_ (Figure 9 inset I) revealed the extreme behavior of these values in the range 0.10 < *x* < 0.20, where the transition from (P) to (L) structure occurs. It is associated with a decreasing *T*_*C*_ to almost room temperature and lower in this concentration range (Figure 9 inset II).

**Figure 10:**
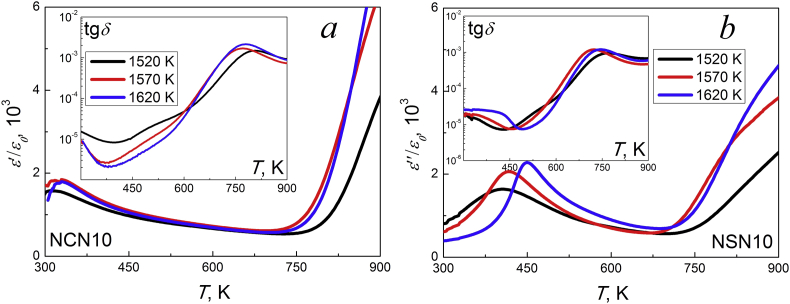
Temperature dependences of *ε'/ε*_*0*_ and *tgδ* at 100 kHz of the NCN10 (a) and NSN10 (b) ceramics obtained at various *T*_*Sin*_.

**Figure 11:**
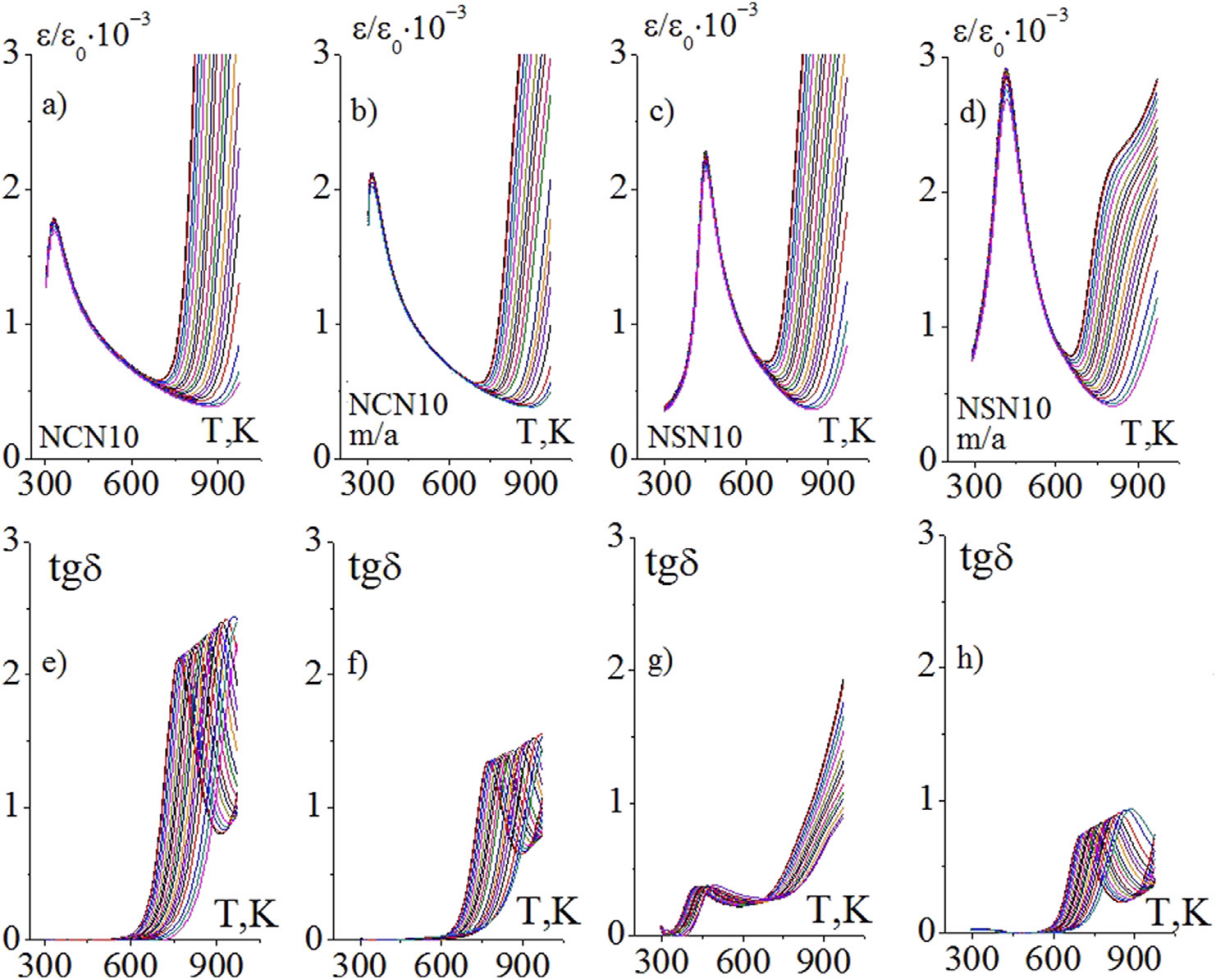
Temperature dependences of *ε'/ε*_*0*_ and *tgδ* of the non mechanoactivated NCN10 (a, e), mechanoactivated NCN10 (b, f), non mechanoactivated NSN10 (c, g) and mechanoactivated NSN10 (d, h) ceramics obtained at *T*_*Sin*_ = 1620 K.

Figures 10 and 11 make it possible to evaluate how technological procedures affect the dielectric characteristics of ceramics from the perovskite zone. In the NCN10 ceramics (Figure 10a) change in the sintering temperature did not significantly affect the dependences *ε'/ε*_*0*_(*T*) and tg*δ*(*T*). However, in NSN10 (Figure 10b), increasing *T*_*Sin*_ shifted *T*_*C*_ to the high temperature region and reduced phase transition blurring. In both systems, mechanically activated samples have a lower Curie temperature and dielectric loss tangent. It seems difficult to assess the influence of technological regulations on the dielectric spectra of samples from the layered region of the phase diagram (NCN25 and NSN25) due to the character of the changes in *ε'/ε*_*0*_ and tg*δ* in the research temperature range of 300÷900 K (continuous increasing dielectric constant and the absence of extrema).

**Figure 12:**
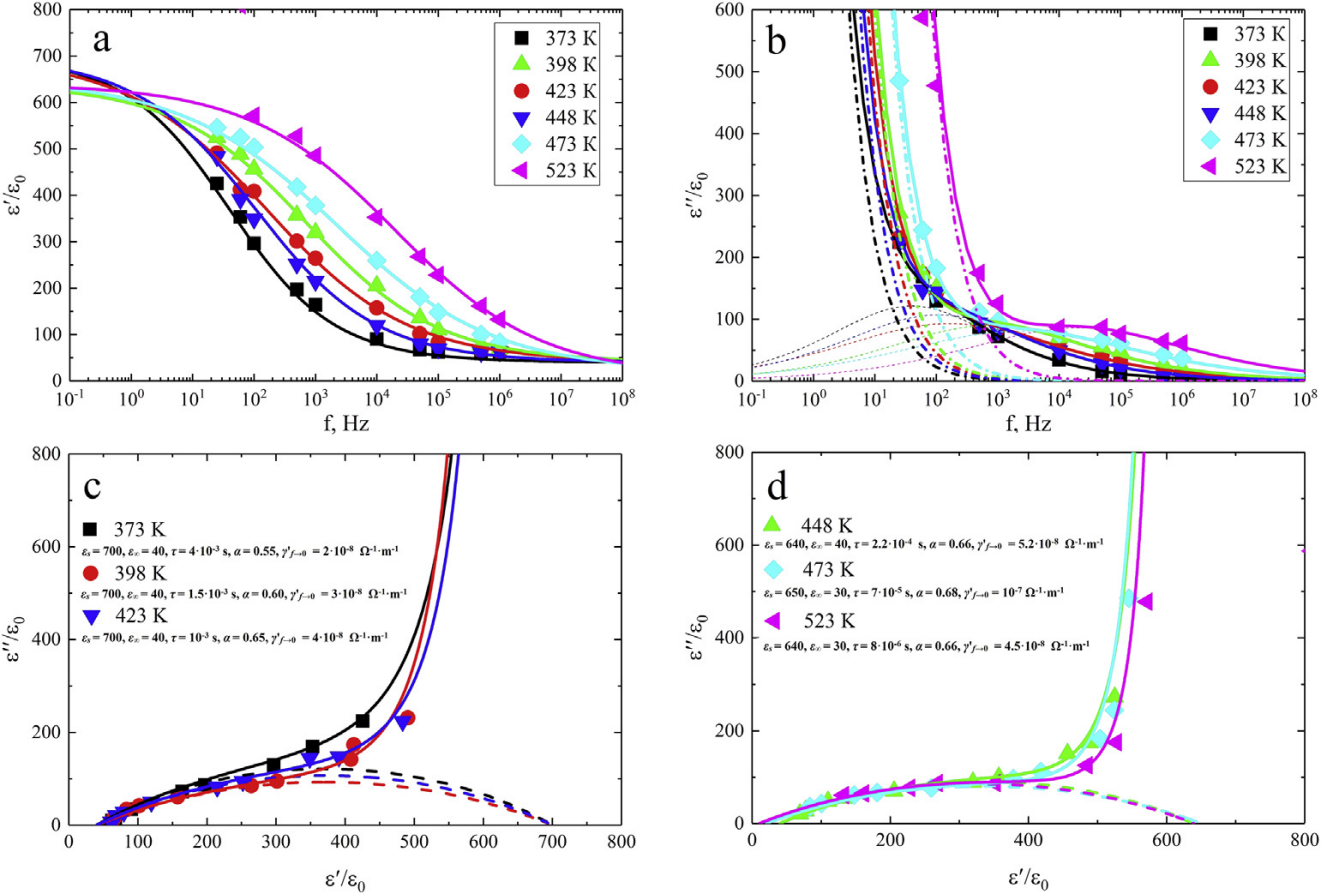
Dependences of *ε*'/*ε*_0_(*f*) (a), *ε*''/*ε*_0_(*f*) (b) and *ε*''/*ε*_0_ (*ε*'/*ε*_0_) (c, d) in the 0.7NaNbO_3_ – 0.3Ca_2_Nb_2_O_7_ solid solutions at 370–520 K (markers). The solid lines correspond to the calculation results for the case taking into account the contribution of the singular term. The dashed lines correspond to the calculation results without taking into account the contribution of the singular term.

In some samples of NCN solid solutions from the concentration range *x* > 0.20, frequency-dependent maxima were observed in the temperature-frequency dependences of the real and imaginary parts of the complex permittivity. These maxima shifted to the high temperatures region and decreased in magnitude as the frequency of the measuring field increased. Figure 12 shows, as an example, the dependences *ε*'/*ε*_0_(*f*), *ε*''/*ε*_0_(*f*) and *ε*''/*ε*_0_ (*ε*/*ε*_0_') at *T* = (370 ÷ 520) K in NCN solid solutions with *x* = 0.3.

Studied spectra show the formation of plateaus *ε*'/*ε*_0_(*f*) at low and high frequencies and maxima of *ε*''/*ε*_0_(*f*), which are most noticeable in the high frequency region as the temperature elevates. In this case, the shape of dependence *ε*''/*ε*_0_ (*ε*'/*ε*_0_) (arc of a circle whose center shifted down relative to the ordinate axis) indicates that the observed relaxation belongs to the non Debye type. This type of relaxation suggests the contribution of through conductivity to the dielectric response of the studied samples. Moreover, this contribution can be significant, as previous studies show [24]. A theoretical description of the temperature-frequency dependences of the real and imaginary parts *ε*∗/*ε*_0_ in the studied frequency range was carried out in the framework of the linear dielectric model with various relaxation time distribution functions. Approximation of the experimental spectra *εʹ(f)* and *εʺ(f)* (Figure 12a and 12b, respectively) was carried out according to formulas (2, 3, 4):

(2) $$\varepsilon^{\text{′}}=\varepsilon_{\infty }+\left(\varepsilon_{s}-\varepsilon_{\infty }\right)\int_{\text{0}}^{\infty }\frac{\text{f}\left(\tau \right)d\tau }{1+{\left(\omega \tau \right)}^{2}}$$

(3) $$\varepsilon^{\text{″}}=\left(\varepsilon_{s}-\varepsilon_{\infty }\right)\int_{\text{0}}^{\infty }\frac{\omega \tau \text{f}\left(\tau \right)d\tau }{1+{\left(\omega \tau \right)}^{2}}$$

(4) $$\int_{\text{0}}^{\infty }\text{f}\left(\tau \right)d\tau =1$$

where *ε*_*s*_ and *ε*_*∞*_ are the static and high-frequency permittivities, respectively. We used different types of distribution functions of non-interacting relaxators (delta function (Debye distribution), equiprobable distribution, Cole-Cole, Davidson-Cole), however, the best approximation of the experimental results was achieved using the Cole - Cole distribution function of the relaxation times *f*(*τ*) (5):

(5) $$\text{f}\left(\tau \right)=\frac{\sin\left(\alpha \pi \right)}{2\pi \text{ch}\left[\left(1-\alpha \right)\ln\left(\tau {\text{f}}_{0}\right)\right]-\cos\left(\alpha \pi \right)}$$

where *α* is the exponent parameter which takes a value between 0 and 1, allows describing different spectral shapes. To eliminate the influence of through conductivity the singular term (*γ*′_*f*→0_/(2π*fε*_0_)), associated with the contribution of the through conductivity, was taken into account at the corresponding frequencies. The approximation results are presented concurrently on Figure 12(c, d, dashed lines).

High values of the parameter *α* indicate a wide spectrum of the distribution of relaxation times. Similar results were obtained for other (1–*x*)NaNbO_3_ – *x*Ca_2_Nb_2_O_7_ solid solutions from the concentration range *x* > 0.2.

Maxwell-Wagner polarization and the corresponding relaxation can be the basis of the physical model describing the observed phenomenon. This type of polarization often called “interlayer”, is manifested, in particular, in an electrically inhomogeneous matrix medium consisting of cells of approximately isodiametric ceramic grains (crystallites), which are surrounded by thin layers with high [25] or low [24, 26] conductivity and *ε∗* different from that of the grains. Observed in this work large variety of grain types, often having different shape and characteristics, leads to the appearance of interlayer polarization and dielectric relaxation in the studied objects.

## 4 Conclusions

Solid solutions of the binary systems (1 – *x*)NaNbO_3_ – *x*Ca_2_Nb_2_O_7_ and (1 – *x*)NaNbO_3_ – *x*Sr_2_Nb_2_O_7_ with non-isostructural extreme components were prepared by solid-phase reaction technique using conventional ceramic technology. Phase diagrams of both systems at room temperature have been determined in the perovskite area. It was shown that this area contains two concentration regions with the different crystal structure and the morphotropic phase boundary between them. The effect of technological regulations on the microstructure and dielectric responses of the NCN and NSN ceramics was studied. It was found that mechanical activation leads to a slight loosening of the grain structure of ceramics, a shift in the phase transition point to the low-temperature region, and a decrease in dielectric loss. Increasing *T*_*Sin*_ leads to enlargement of ceramic grains, and also, in the case of NSN from the perovskite region of the phase diagram, to increase in the Curie temperature and a decreasing phase transition blurring. Using both variations in the sintering temperature and mechanical activation makes it possible to obtain ceramics with a high density, which is a very good result for conventional ceramic technology. It was established that in several samples from layered zones of the phase diagram of both systems, the Maxwell-Wagner polarization and the corresponding relaxation are present. What manifested itself in the presence of frequency-dependent maxima in the dependences *ε'/ε*_0_(*T*) and tg*δ*(*T*). It was established that the anomalies, which are the consequence of the non-Debye dielectric relaxation and are shifted to the high temperature region with an increase in the frequency of the measuring electric field, are formed in the (1 – *x*)NaNbO_3_ – *x*Ca_2_Nb_2_O_7_ ceramics from the concentration range *x* > 0.2 on the *ε'/ε*_0_(*T*) and *ε*''*/ε*_0_(*T*) dependences The most probable cause of the revealed process can be described by Cole - Cole distribution function Maxwell–Wagner relaxation with the wide spectrum of the distribution of relaxation times in the crystallite–interlayer heterogeneous system. The data obtained should be used in the development of new materials based on calcium and strontium pyroniobates.

## Declarations

### Author contribution statement

Jaroslav Y. Zubarev, Nikita A. Boldyrev, Anatoly V. Pavlenko: Performed the experiments; Wrote the paper.

Shun-Hsyung Chang, Chitsan Lin, Ivan A. Parinov: Analyzed and interpreted the data.

Alexander V. Nazarenko, Alexander V. Nagaenko, Yuri I. Yurasov: Performed the experiments.

Ilya A. Verbenko: Contributed reagents, materials, analysis tools or data.

Larisa A. Reznichenko: Conceived and designed the experiments.

### Funding statement

This work was supported by the 10.13039/501100004663Ministry of Science and Technology, Taiwan (MOST105-2923-E-022-001-MY3, MOST108-2221-E-992-052-MY2, MOST 108-2221-E-992-026) and the 10.13039/501100003443Ministry of Education and Science of the Russian Federation (BAZ 0110/20-3-07IF).

### Competing interest statement

The authors declare no conflict of interest.

### Additional information

No additional information is available for this paper.
